# Correction: Magnetic coupling between Fe(NO) spin probe ligands through diamagnetic Ni^II^, Pd^II^ and Pt^II^ tetrathiolate bridges

**DOI:** 10.1039/d3sc90183a

**Published:** 2023-09-21

**Authors:** Manuel Quiroz, Molly M. Lockart, Shan Xue, Dakota Jones, Zachary Martinez, Yisong Guo, Brad S. Pierce, Kim R. Dunbar, Michael B. Hall, Marcetta Y. Darensbourg

**Affiliations:** a Department of Chemistry, Texas A &M University College Station Texas 77843 USA marcetta@chem.tamu.edu; b Department of Chemistry & Biochemistry, Samford University Birmingham Alabama 35229 USA; c Department of Chemistry, Carnegie Mellon University Pittsburgh Pennsylvania 15213 USA; d Department of Chemistry & Biochemistry, University of Alabama Tuscaloosa Alabama 35487 USA

## Abstract

Correction for ‘Magnetic coupling between Fe(NO) spin probe ligands through diamagnetic Ni^II^, Pd^II^ and Pt^II^ tetrathiolate bridges’ by Manuel Quiroz *et al.*, *Chem. Sci.*, 2023, **14**, 9167–9174, https://doi.org/10.1039/D3SC01546G.

The authors regret that a co-author, Zachary Martinez, was omitted in error from the author list of the published article.

The Royal Society of Chemistry apologises for these errors and any consequent inconvenience to authors and readers.

## Supplementary Material

